# Teachers’ conflicts in implementing comprehensive sexuality education: a qualitative systematic review and meta-synthesis

**DOI:** 10.1186/s41182-023-00508-w

**Published:** 2023-03-28

**Authors:** Fumiko Shibuya, Crystal Amiel Estrada, Dian Puspita Sari, Rie Takeuchi, Hirono Sasaki, Cut Warnaini, Saki Kawamitsu, Hamsu Kadriyan, Jun Kobayashi

**Affiliations:** 1 Department of Global Health, Graduate School of Health Sciences, University of the Ryukyus, 207 Uehara, Nishihara, Okinawa 903-0215 Japan; 2 Japanese Consortium for Global School Health Research, 207 Uehara, Nishihara, Okinawa 903-0215 Japan; 3 Department of Environmental and Occupational Health, College of Public Health, University of the Philippines Manila, 625 Pedro Gil Street, Ermita Manila, Philippines; 4 Faculty of Medicine, University of Mataram, Jalan Pendidikan 37, Mataram, West Nusa Tenggara 83125 Indonesia

**Keywords:** Comprehensive sexuality education, Teachers’ conflicts, Qualitative systematic review, Meta-synthesis

## Abstract

**Introduction:**

Comprehensive sexuality education (CSE) enables children and young people to learn about the cognitive, emotional, physical, and social characteristics of sexuality. Teachers experience conflicts in teaching CSE due to different cultural and religious backgrounds. This qualitative systematic review aimed to describe the conflicts experienced by teachers in the implementation of CSE in schools. Furthermore, this study aimed to identify the causes of conflict among teachers in implementing CSE.

**Methods:**

This article focused on teachers’ conflicts in implementing CSE from 2010 to 2022. Online bibliographic databases, such as PubMed, Web of Science, and ERIC, were used to search for relevant articles. The following search term was used: *Teacher, Comprehensive Sexuality Education, and Conflict*.

**Results:**

A total of 11 studies were included in the review. All 11 studies were conducted in countries with a predominantly Christian population. The majority of the studies were conducted in Africa. The study respondents included teachers, school principals, and school coordinators. The studies identified that CSE implementation is related to multiple conflicts, depending on the context of the country. Five themes on the causes of conflict emerged from the thematic meta-synthesis: (1) Hesitancy in talking about sex education among teachers due to the cultural and religious context; (2) non-integration of traditional sex education into comprehensive sexuality education, (3) fostering effective facilitation of CSE among teachers, (4) determining the appropriate age to start sex education, and (5) roles of stakeholders outside the school.

**Conclusions:**

This qualitative systematic review and thematic meta-synthesis highlighted several conflicts among teachers in CSE implementation. Despite the teachers having a perception that sex education should be provided, traditional sex education has not yet transformed to CSE. The study findings also emphasize the need to identify the teacher’s role in CSE implementation. The thematic meta-synthesis also strongly reflected the context of Christianity in Europe and Africa; thus, further research on the religious context in other regions is needed.

## Introduction

School health education may lead to better health outcomes with intervention methods in the school setting. The Health Promoting School concept was recommended by the World Health Organization (WHO) and other United Nations (UN) partners to enhance health and the ability to acquire knowledge, skills, attitudes, and values among children and adolescents at school [1]. Efforts in health promotion are associated not only with increasing knowledge of healthy attitudes and behavior but also with a slight reduction in active sexual behavior and mental health issues among adolescents [2]. In particular, for adolescents, educators should provide life skills education, including education on sexual reproductive health and rights, in cooperation with parents and the community [3].

The United Nations Educational, Scientific and Cultural Organization (UNESCO) advocated for comprehensive sexuality education (CSE) in 2009 to help young people make responsible choices in relation to appropriate sexual behavior by acquiring the right scientific knowledge and skills according to their age and culture [4]. CSE is essential to enhance adolescent health because of increased sexual and reproductive health issues globally. However, there were some challenging issues with the implementation of the CSE due to religious and cultural backgrounds in the society and community, because school health policy on sexual education was not recommended in some countries [5]. According to the CSE status report in 2021, 85% of 155 countries surveyed have policies or laws relating to sex education [6]. Furthermore, most of the countries indicated that they have some curriculum based on the policies or laws in each country. However, this report evaluated that curricula were not enough to deliver sex education effectively. Teachers were also provided with training, but many still did not feel confident to deliver sex education to students [6]. Teaching sex education is strongly influenced by social norms and experiences from personal views, leaving teachers often feeling uncomfortable or defensive about it [7]. Moreover, some of the barriers to the implementation of CSE were impacted by culture and religion, and it was associated with the school context and community [8]. Therefore, to promote the implementation of CSE as part of school health policies, it is necessary to consider the cultural and religious background of each country.

Cultural and religious factors influence the implementation of CSE. Teachers’ confidence in CSE implementation is shaken by the cultural and religious backgrounds of their communities and by fears of negative effects, such as encouraging students to engage in unhealthy sexual behavior. Sexual topics are related to the traditional culture and local situation [9]; thus, consideration of these factors is crucial for the promotion of CSE. Moreover, it is considered that the guidance of sexuality education is strongly related not only to educational institutions but also to policies and traditional values of society [10]. Unfortunately, previous studies relevant to CSE were mostly conducted in the context of predominantly Christian regions [9, 10] and there is a paucity of studies focusing on the context of other religions. According to the systematic review of CSE in low-and-middle-income countries in 2022, the findings of this review indicated that multiple factors such as social, economic, cultural, and political influenced the implementation of CSE and integration into the educational systems [11]. Furthermore, quantitative studies conducted previously have not yet identified the common cultural and religious factors affecting school-based sexuality education implementation.

In psychology, conflict is defined as the arousal of two or more strong motives that cannot be solved together [12]. Conflict can be defined as an expression of hostility, negative attitudes, antagonism, aggression, rivalry, or misunderstanding. Moreover, it is associated with situations that involve contradictory or irreconcilable interests between two opposing groups. In psychology, the theory of conflict was advocated by Kurt Lewin in 1935 [13]. This qualitative systematic review focused on the conflicts experienced by teachers in the implementation of CSE in the school setting.

This qualitative systematic review aimed to explore teachers’ experiences of conflict in the implementation of CSE in schools and to identify the causes of conflict to overcome the challenges. The following review question was considered: *What conflicts are experienced by teachers in the implementation of CSE in the school setting?* Moreover, the systematic review aimed to answer the following questions:

1. *Causes of conflict*: What types of conflict occurred in implementing CSE among teachers?
2. *Overcoming conflict*: According to the causes of conflict (primary outcome), how do teachers overcome the conflicts in implementing CSE?

## Methods

This systematic review adopted a meta-synthesis approach and adhered to the ENTREQ and PRISMA guidelines [14–17]. The review protocol was registered with PROSPERO (registration number: CRD42022353313). The qualitative systematic review was conducted in accordance with the Preferred Reporting Items for Systematic Reviews and Meta-Analyses (PRISMA) protocol [17]. The included studies were evaluated using the Joanna Briggs Institute Critical Appraisal Checklist [18, 19].

### Search strategy

The study focused on conflict among teachers in the implementation of CSE from 2010 to 2022. The search was limited to the period of 2010–2022, because CSE was promoted in 2009. The search strategy was constructed as follows: Search #1 Target population; Search #2 comprehensive sexuality education; Search #3 Implementation; and Search #4 Setting (Table 1). The basic formula utilized was as follows: (teacher*) AND (“comprehensive sex education” OR “comprehensive sexual education” OR “comprehensive sexuality education” OR “sex education” OR “sexual education” OR “sexuality education”) AND (“conflict*” OR “challenge*” OR “factor*” OR “experience*” OR “perception*” OR “attitude*”) AND (“school*” OR “primary school*” OR “secondary school*” OR “junior high school*” OR “senior high school*” OR “high school*”).

**Table 1:** Keywords and the combinations used in the search strategy

Search strategy		
	Characteristic	Search terms combined with AND
Search #1	Target population	teacher*
		AND
Search #2	Comprehensive sexuality education	comprehensive sex education OR comprehensive sexual education OR comprehensive sexuality education OR sex education OR sexual education OR sexuality education
		AND
Search #3	Implementation	conflict* OR challenge* OR factor* OR experience* OR perception* OR attitude*
		AND
Search #4	Setting	school* OR primary school* OR secondary school* OR junior high school* OR senior high school* OR high school*

### Eligibility (inclusion and exclusion) criteria

The title and abstract of the results generated from the searched database were screened using the following inclusion criteria:

1. Publication period: studies published in a peer-reviewed journal and written in English from 1 January 2010 to 1 August 2022.
2. Participants: teachers at primary schools, secondary schools, and senior schools.
3. Study objective: to review the relevance of teachers’ conflict in implementing CSE.
4. Study design: all qualitative study designs, including mixed methods studies.
5. Location and language: focused on all countries and written in English.

Inclusion criteria were considered to identify the justification of the qualitative systematic review from five perspectives (Table 2). Teachers who did not work at primary or secondary schools were excluded from the study.

**Table 2:** Summary of the inclusion criteria

Subject	Criteria	Justification
Publication period	Conducted from 2011 to 2022	The search was limited to the period of 01/01/2010 to 01/08/2022 because CSE was advocated in 2009. Studies published before 2011 will be excluded, to ensure the review examines current practice and challenges
Participants	Focused on teachers who work in the school	This qualitative systematic review should identify what teachers’ conflicts are based on their experiences and perceptions in the school. Therefore, we targeted teachers who work in the school to clarify their conflicts when implementing CSE
Study objectives	Relevant to teachers’ conflicts in implementing CSE or Sex Education	CSE covers various topics, such as relationships, rights, understanding of gender, and violence based on the components advocated by UNESCO. However, most of the studies do not include comprehensive content. Therefore, this review included traditional “Sex Education” on the search formula to find a study on the implementation of CSE and sex education
Study design	All Qualitative study (Includes a mixed methods study)	This review focused on teachers' conflict or experience, and perception of the implementation of the CSE, which is most appropriately answered through a qualitative study or a mixed methods study. Therefore, any purely quantitative study was excluded through the screening process
Location and Language	All country, and published in the English Language	We targeted all countries; however, there are limitations in translating studies published in languages other than English and were thus excluded from the review

### Information sources

Online bibliographic database search was conducted. PubMed, Web of Science, and ERIC were used to search for relevant articles. The search formula was adapted to each database style. No geographic limit was applied to the search.

### Study selection

First, duplicates were removed, and articles published outside 2010 to 2022 were excluded. Then, two researchers (FS and SK) independently screened the selected articles, first by title and abstract. Next, full-text articles were screened based on the inclusion criteria of the review protocol. Eligibility was negotiated by consensus among co-researchers at each stage of the screening process.

### Quality appraisal

All titles and abstracts that were identified through the literature search were included in the primary review process. The quality of the included studies was independently assessed by two researchers with master’s degrees (FS and SK), according to the JBI Critical Appraisal Checklist for Qualitative Research [19]. The JBI Critical Appraisal Checklist contained ten questions addressing the possibility of bias in research design, conduct, or analysis. The studies were appraised using the checklist and were categorized into three. First, the studies which fulfilled the inclusion criteria that have a low level risk of bias were identified as A level. Second, the studies which partially fulfilled the inclusion criteria with a moderate risk of bias were identified as B level. Finally, the studies which did not satisfy the inclusion criteria and had a high risk of bias were categorized as C level. Articles that reached A or B levels were included in the meta-synthesis. The results from the selected studies were analyzed through a thematic analysis to synthesize all of the relevant studies that included a qualitative systematic review. Meaning units/supporting quotations were then extracted from the results of the included studies and analyzed to conduct thematic meta-synthesis.

### Data synthesis

The thematic-meta synthesis was conducted in three stages, as described by Thomas and Harden in 2008 [19]: (i) line-by-line coding; (ii) developing descriptive themes; and (iii) generating analytic themes.

i) Coding
  In the first stage of inductive coding, relevant qualitative data were extracted from the results of each study that included meaning units/supporting quotations.
ii) Descriptive theme
  In the second stage of the inductive thematic analysis, descriptive themes were created to systematize the codes.
iii) Analytic themes
  In the third stage of deductive thematic analysis, the descriptive themes were synthesized into analytic themes according to Lewin’s theory of conflict [13].

### Procedure of the meta-synthesis

The data extraction and meta-synthesis were conducted in three stages: (i) coding; (ii) development of descriptive themes; and (iii) creation of analytic themes.

i) Coding
  The principal investigator (FS) independently conducted the coding process under the supervision of two researchers (RT and JK). All extracted codes from the selected studies were compiled in an analysis sheet. The principal investigator (FS) and two researchers (RT and JK) reviewed the extracted codes to ensure that these were based on the data from the selected studies.
ii) Development of descriptive themes
  First, the principal investigator (FS) developed the descriptive themes based on the extracted codes under the supervision of two researchers (RT and JK). Next, the principal investigator (FS) and two researchers (RT and JK) ensured that the extracted descriptive themes were based on the codes that were recorded in the analysis sheet.
iii) Creation of analytic themes
  First, the principal investigator (FS) created the analytical themes and sub-themes based on the developed descriptive themes under the supervision of two researchers (RT and JK). Second, the principal investigator (FS) and two researchers (RT and JK) ensured and elaborated the analytical themes that were based on the analysis sheet and were created to synthesize all findings from the studies. Finally, the principal investigator (FS) and five researchers (CE, DPS, RT, HK, and JK) discussed the findings and resolved disagreements through consensus to finalize the validity of the extracted codes, descriptive themes, and analytical themes.

## Results

### Screening process

**Fig. 1:**
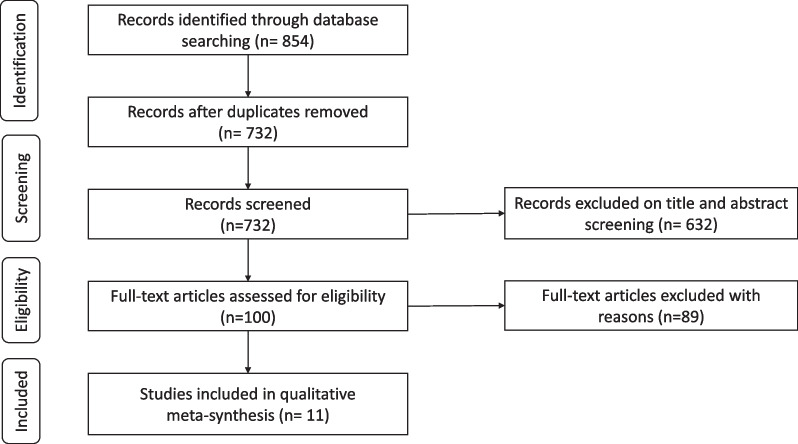
PRISMA flow chart of the review (PRISMA)

A total of 854 studies were identified during the screening process from the three databases: PubMed (*n* = 166), Web of Science (*n* = 335), and ERIC (*n* = 353). After removing the duplicates, 732 studies remained. Six hundred thirty-two citations were excluded after the title and abstract screening, leading to the retention of 100 potentially relevant articles. Finally, 11 studies were included in the meta-synthesis. The search outcomes are summarized in the PRISMA flowchart of the review in Fig. 1 [17].

### Characteristics of the included studies

The characteristics of the included studies are summarized and presented in Table 3. The majority of the studies included in the review took place in Africa. Of the 11 studies, nine were qualitative and two were mixed methods studies. Regarding the participants’ characteristics, 11 studies targeted teachers and included school principals and school coordinators. Participants included teachers who teach sexuality education in the school setting. Most of the studies focused on the context of Christianity, having been conducted in predominantly Christian regions and involving Christian participants.

**Table 3:** Characteristics of the studies included in the systematic review

No	Citation	Country	Study design	Participants	Study aim	Main findings
1	Le Mat et al. (2021) [20]	Ethiopia	Qualitative study (content analysis)	Total 56 participants, included 12 teachers	To improve understanding of the ways in which teachers enact and re-contextualise CSE policy, and their reasons for doing so To understand teacher enactment, we attend not only to what teachers do, but also to teachers’ interpretations of the policy	The paper highlights that while CSE teachers were typically conceptualised as ‘facilitators’ of the CSE initiative presented here, in practice teachers seemed to perform what might be understood as ‘activist’ roles within and beyond the classroom and school
2	Ocran. (2021) [21]	Ghana	Qualitative case study	Total 13 participants; included Municipal School Health Education Coordinator, head teachers, school-based coordinators, and students at junior high schools	To investigate the training and support offered to teachers on the delivery of sex education in three contrasting Junior High Schools in Ghana, the attitudes and approaches to the delivery of sex education, and the response of students to teacher delivery of sex education	Findings suggest that students underwent training sessions in sex education through classroom-based learning, and extracurricular activities such as school health clubs, talks by nurses, and also talks by teachers during early morning school worship Findings also show that although teachers in all schools underwent the same level of training for sex education, they exhibited different attitudes to teaching
3	De Hass and Hutter. (2019) [22]	Uganda	Qualitative study (grounded theory)	40 sexuality education teachers from 16 secondary schools	To obtain a better understanding of teachers’ cultural values and beliefs, the conflicts they may experience, and how these relate to their experienced level of comfort teaching comprehensive sexuality education within the Ugandan context	This study partly confirms those findings but also shows that teachers can feel conflicted about the type of messages their students need and can feel vulnerable to adopting more comprehensive approaches within a school system that expects the to teach abstinence-only
4	Achora et al. (2018) [23]	Uganda	Grounded theory design	11 teachers participated in individual interviews in four rural primary schools	To explore the experiences and perceptions of teachers and adolescents (12–16 years) of school-based sexuality education in rural primary schools in Uganda	Findings from the study have shown that adolescents received abstinence-only information as a method to protect themselves against STIs/HIV and to continue attending school The findings also demonstrated that sexuality education was taught selectively, since it was considered not to be relevant for children at primary level
5	De Hass and Hutter. (2020) [24]	Uganda	Qualitative study (grounded theory)	40 sexuality education teachers from 16 secondary public (n=7) and private (n=9) schools	To gain a better understanding of teachers’ professional identities in the context of providing school-based sexuality education in Uganda, and of how these identities motivate teachers to provide school-based sexuality education	Five cultural schemas of professional identity were found: (i) upholder of ethics and regulations; (ii) authority figure; (iii) counsellor and guide; (iv) role model; and (v) guardian Teachers’ cultural schemas of professional identity motivated them to adhere to moral discourses of abstinence and sexual innocence
6	Louw. (2017) [25]	South Africa	Qualitative study (thematic analysis)	Total 78 participants; included 68 teachers and 10 school staffs at special needs schools	To focus specifically on teachers and school staff employed at Special Needs Schools in the Western Cape Province to explore their perspectives and views on teaching sexuality and HIV and AIDS education to learners with disabilities	This study holds that the impact of environmental factors as it relates to societal attitudes towards youth with disabilities and their sexuality is potentially harmful to developing a positive sexuality
7	Håkansson et al. (2020) [26]	Kenya	Mixed methods study	15 teachers at a secondary school	To explore attitudes related to abortion and contraceptive use among secondary school teachers and student peer-counsellors in a low-resource setting in western Kenya	This study showed that adolescent girls associated with abortion and contraceptive use face social judgements and discrimination by secondary school teachers and fellow students in Kenya
8	Rijsdijk et al. (2014) [27]	Uganda	Mixed methods study	Of the 24 teachers from the intervention schools, eight were selected for in-depth interviews	The purpose of this mixed-methods study was to examine factors associated with dose delivered (number of lessons implemented) and fidelity of implementation (implementation according to the manual), as well as to identify the main barriers and facilitators of implementation	Teachers’ beliefs/attitudes towards sexuality of adolescents, condom use and sex education were found to be important socio-cognitive factors intervening with full fidelity of implementation
9	De Hass and Hutter. (2022) [28]	Uganda	Qualitative study (grounded theory)	40 sexuality education teachers from 16 secondary schools	Cultural schema theory was used to explore teachers’ personal experience of the onset of sexual activity and explain how sexuality education teaching is influenced by such experiences	Findings show that while teachers’ personal experience of sexual initiation did not directly align with the content of their messages, due to the centrality and evocative function of these schemas these experiences strengthened teachers’ motivation to teach sexuality education because they enabled them to empathise with students
10	Plaza-Del-Pino et al. (2021) [29]	Spain	Descriptive Qualitative study	15 active primary school teachers in four public primary schools	To explore the perspective of primary school teachers regarding Sexual Education in schools in Spain	Primary school teachers conclude that SE in schools has an excessively preventative approach Nonetheless, they recognise an attempt to implement a more comprehensive SE that includes affective sexual education and sexuality as a human right
11	Zulu et al. (2019) [30]	Zambia	Case study	18 teachers from six schools in Nyimba district	To investigate teachers' experiences with the implementation of the CSE curriculum in the Zambian context	This study’s findings revealed that the lack of clarity in the CSE framework, on how to integrate CSE teaching into existing subjects, coupled with contextual challenges, left teachers involved in CSE with a great room for discretion

### Study selection quality appraisal

The methodological quality of the review was assessed according to the Joanna Brigs Institute Critical Appraisal Checklist for Qualitative Research [18, 19]. The results of the study quality appraisal are shown in Table 4. All included studies described the philosophical perspective. The background and potential influence of the researcher were not addressed in most of the studies.

**Table 4:** Quality appraisal of the included studies (Joanna Briggs Institute Critical Appraisal Checklist)

No	Study	Q1	Q2	Q3	Q4	Q5	Q6	Q7	Q8	Q9	Q10	Level
1	Le Mat et al. (2021) [20]	Y	Y	Y	U	U	N	N	U	N	U	**B**
2	Ocran. (2021) [21]	Y	Y	Y	Y	Y	N	N	Y	Y	Y	**B**
3	De Hass and Hutter. (2019) [22]	Y	Y	Y	Y	Y	Y	Y	Y	Y	Y	**A**
4	Achora et al. (2018) [23]	Y	Y	Y	Y	Y	N	Y	Y	N	Y	**B**
5	De Hass and Hutter. (2020) [24]	Y	Y	Y	Y	Y	N	N	Y	Y	Y	**B**
6	Louw. (2017) [25]	N	Y	U	Y	Y	N	Y	Y	Y	Y	**B**
7	Håkansson et al. (2020) [26]	Y	Y	Y	U	Y	N	Y	Y	N	Y	**B**
8	Rijsdijk et al. (2014) [27]	Y	Y	Y	Y	Y	N	N	Y	Y	Y	**B**
9	De Hass and Hutter. (2022) [28]	Y	Y	Y	Y	Y	N	N	Y	Y	Y	**B**
10	Plaza-Del-Pino et al. (2021) [29]	Y	Y	Y	Y	Y	N	N	Y	Y	Y	**B**
11	Zulu et al. (2019) [30]	Y	Y	Y	Y	Y	N	N	Y	Y	Y	**B**

Y: yes; N: no; U: unclear; NP: not applicable

Q1. Is there congruity between the stated philosophical perspective and the research methodology?

Q2. Is there congruity between the research methodology and the research question or objectives?

Q3. Is there congruity between the research methodology and the methods used to collect data?

Q4. Is there congruity between the research methodology and the representation and analysis of data?

Q5. Is there congruity between the research methodology and the interpretation of results?

Q6. Is there a statement locating the researcher culturally or theoretically?

Q7. Is the influence of the researcher on the research, and vice-versa, addressed?

Q8. Are participants, and their voices, adequately represented?

Q9. Is the research ethical according to current criteria or, for recent studies, and is there evidence of ethical approval by an appropriate body?

Q10. Do the conclusions drawn in the research report flow from the analysis, or interpretation, of the data?

### Thematic meta-synthesis

The studies included in the meta-synthesis identified multiple conflicts CSE implementation depending on the country’s context. The studies focusing on conflicts among teachers in implementing CSE were conducted in seven countries, namely: Ethiopia, Ghana, Kenya, South Africa, Uganda, Zambia, and Spain. Five themes were generated from the thematic meta-synthesis (Table 5): (1) Hesitancy in talking about sex education among teachers due to the cultural and religious context; (2) Non-integration of traditional sex education into comprehensive sexuality education; (3) Fostering effective facilitation of CSE among teachers; (4) Determining the appropriate age to start sex education; and (5) Roles of stakeholders outside the school.

**Table 5:** Analytical and descriptive themes

Analytical theme	Sub-theme (ST)	Descriptive theme	Supporting quotations/meaning units
Theme 1: Hesitancy in talking about sex education among teachers due to the cultural and religious context	ST 1-1: The topic of Sexual and Reproductive Health (SRH) is a still taboo	A teacher hesitated to talk about reproductive health to students at school	*I think this problem [to not speak openly about reproductive health]... culture, the society’s culture. [CSE] is a rich mission to minimise this. (D2, school4, FGD with teachers, participant5, male)* [20]
		Teacher’s belief that students still cannot make the right decisions, so they need directions from teachers to choose the right way	*As [teachers] we believe that giving a free choice to these very young children is dangerous. SRH is sensitive issue that determines the future life of the youth. So as a teacher, we need to show them the right way. Just discussing the options and leaving the choice to them is dangerous because they are kids who do not know what is right and wrong (Validation workshop, teacher)* [20]
		Lack of interest to facilitate CSE implementation	*According to me, the main problem is that the school administration lacks interest to facilitate [CSE] like that of regular programmes. They have no interest to facilitate these issues. (D2, school4, FGD with teachers, teacher3, male)* [20]
		The teacher felt shy to teach about sex education, but it's a huge challenge	*Some of the teachers feel shy to teach about sex education and I think this is a huge challenge. (School-based Coordinator, School Two)* [21]
		Sexuality education has not been reinforced at home because it was not opened topics	*Firstly, the facts discussed on this topic are not reinforced at home. Therefore, it is easily forgotten. Sexuality is also not an open topic at home (grade 12 teacher, black female, 10 to 20 years teaching experience)* [25]
		Teaching sexuality education in a school is taboo due to culturally inappropriate	*The controversy is also about the place, where such information is delivered from not being culturally appropriate, it’s taboo to teach sexuality education in a school (IDI, Teacher 11)* [30]
	ST 1-2: Concerns that sexuality education might encourage unhealthy curiosity and experimenting sexual behaviors	Some of the parents have not yet acknowledged talking about sexuality at school because they felt about sexuality education becomes 'sexual awake' to the children	*Some parents do not give permission to the school to talk about sexuality to the children because some parents feel that the teacher will ‘awake’ something in their child and make the child curious to experiment (grade 5 teacher, coloured female, 10 to 20 years teaching experience)* [25]
		Teachers use their knowledge on sexuality to discourage students from engaging in sexual behaviors	*Now, when, eh, I did give a talk to students how… they have a habit of standing in corners and then kissing and then, we call it coupling, boy and girl, huh? And I wanted to discourage them so I got… I talked about HPV, the cancer virus, and all that, and somehow I really scared them into thinking if you kiss, […] you can get cancer and something like that! [chuckles] […] [But] it’s not fair to the students; we should not scare them into doing what we think is right. And another thing what I realised, after that talk, there could have been a change in the first week, but after, these kids are going to go back to their old ways, so… (Female, age 29)* [24]
		The most dangerous one is becoming active in sex matters at the adolescence stage	*Those ones who are just becoming active, in sex matters, adolescence stage, is the most dangerous one. Because if the kid is not well guided, can land into many problems […]. When you’re guiding, you show them the bad, the problems, then you put them right. […] You show them the proper ways […] to live with this future. (Male, age 25)* [24]
		Sexuality education is complicated because of a heavy workload to educate children on the sexual topic	*It’s complicated, we already have a heavy workload to have to then educate children in a topic for which we have not been prepared, to be honest (worried face). (E9)* [29]
	ST 1-3: Concerns/Fear that teacher would lose moral authority and control over the students	The teacher recommended students to practice abstinence	*I normally encourage young girls and boys to abstain from sex because I know the dangers… For us we are victims of what our parents did… So normally when we are teaching some of, eh, these topics, huh? We basically use the Bible … how can … a Christian … how can a youth abstain from sex? (Peter, age 28)* [22]
		Especially young girl has sexual weaknesses and should not expose outside	*I made a resolution: never to have sex with anybody I teach. […] If I choose to […] then I should do it in another school. […] Once I stand before somebody, I always want to command respect before that… young girl […]. I have sexual weaknesses, I should not expose them in the work place. That became my only guiding principle. But still, it did not help me because out there… I would still engage into sex relations with VARIOUS [with emphasis] other people, not necessarily those who were in school. (Male, age 31)* [24]
		Concerns about violating professional code of conduct as a teacher	*Yes, there are times when they ask you something, when […] you are ignorant about it, other times, you may be knowing… but you feel explaining it to them as a teacher, […] it’s like […] you have gone against your professional code of conduct, other times you don’t know the… the language, how to phrase it, such that it does not appear too obscene. (Female, age 35)* [24]
		Respect the authority of teachers at schools as an educator	*In schools, teachers are superior because they are the ones in authority, and the students have to respect authority. […] The academic bit of it… lowers them [students] […] and understand that this one must be […] better than us’ (Female, age 35)* [24]
		The teachers try to maintain hierarchy of respect with their students and this led them to believe their students know nothing about sexuality	*So many of them [teachers]… have maintained that culture of… erm… keeping that distance… from the student. But I think it’s also a deception really to, to begin thinking that you can keep these children young… and you know, innocent, and they should not speak anything! […] They want to maintain that […] hierarchy of respect. […] (Female, age 37)* [24]
		Relationships between students and educators have not been established because teachers are looked at as disciplinarians	*Teachers are looked at as disciplinarians, they are very tough, they are very strict. […] So one is just to command without any question, without any challenge. So you find that, eh, there is no relationship established. (Female, age 37)* [24]
		The teacher fight to maintain their integrity and is afraid of losing the moral authority to control students	*If you have to talk to a Form 1, huh? You have to be very careful about… what you say out because… behind there is integrity. Once somebody loses it as a teacher, then… you have virtually no control over this person… because you’ve lost the moral authority. […] So in a way we fight to maintain that integrity, to allow us chance to address them… on certain issues and earn their trust… (Male, age 30)* [24]
	ST 1-4: Providing information on contraceptive methods is depending teachers’ perceptions and experiences	The teacher applied different teaching about contraception to different audiences based on their faith	*If I’m addressing a congregation of strictly Saved girls and boys, I will not mention a condom… But if I’m talking to these lay people … I would warn them and tell them: [chuckles a bit] ‘You had better use a condom’. Me, I believe it is much safer, because one it will protect, it will protect somebody from … HIV but also pregnancy. (Grace, age 37)* [22]
		Worries/concern that teaching how to use condom will be understood as encouraging sexual behaviors due to autocratic teaching style	*We just tell them: don’t have sex … or use a condom. But so much it is that when you teach a kid how to use a condom, then the kid will go and, and use it! … So … that idea of sharing … information, it’s more of ordering, I think, it’s from the side of the teachers, because we are used here more to give in commands rather than discussing and sharing experiences. (Paul, age 32)* [22]
		Teacher did not teach different methods of contraception as expected from them	*We are expected to discuss types of contraception such as oral contraceptive pill and depo-provera through injectable method, implant, but I do not teach them such things (IDI, Teacher 10)* [30]
			*One thing is, we fear, for example, if you demonstrated how … eh, a condom is used in a secondary setting, we fear that maybe a student … they go and practise [laughs]… So, we prefer that you’d rather keep … a student in the dark, when they don’t know particular things. (Grace, age 37)* [22]
	ST 1-5: Religious and cultural norms as inhibitor in implementing sex education	Sexuality education is still inhibited due to both backgrounds culture and religion, and multiple pressures might be impacted the educator (insulator)	*You may say it at the risk of your job. Because if you do it … consistently, you may be looked at as a person who is spoiling … you need some insulator, something to insulate you from … the culture and the religious and the other … the other pressures that try to inhibit people doing such sexuality education. (Paul, age 32)* [22]
		The teachers are providing sex education based on the Catholic bible at the Catholic-founded school	*Yes, because first of all this is a Christian school, it’s a Catholic founded school, and … those [accepted sexual acts] are the principles we teach in the school … we base our teaching on the Bible… (Vivienne, age 43)* [22]
		The teacher belief that teaching students about morality is their duty to the nation	*We [teachers] also feel … that … we have a duty to the nation… Because then what will be the use of … educating them … [if] you’re sending out … very immoral people, you’re sending out corrupt people … who are very reckless with their lives, so they can’t do anything for the nation at all. (Barbara, age 26)* [22]
		The traditional view of sex as a secrecy that only being discussed among married people and not for the young people	*You know, in our culture … sex is supposed to be kept … a secret, such that these young people are not supposed to know anything about it… Traditionally, things related to sex were not supposed to be … to be exposed to the young people until they have reached the age of marriage. (Jane, age 32)* [22]
	ST 1-6: Abstinence education as inhibitor of CSE implementation	The teacher hopes to share the belief of the benefit of abstinence education with students	*I’ve seen the good things [...] that come from abstinence and at least I would like to share them with my students because apart from mending my students, they are my friends. So, I would like to tell them that erm... if one abstains, [...] you’re free like a bird, [...] you do anything you want. (Sandra, age 22)* [28]
		The main message is abstinence when teaching contraception at school	*In the school setting, when I teach about preventing pregnancy, the main message is only about abstinence (IDI, Teacher 16)* [30]
		Emphasized promoting abstinence education to avoid the vicious cycle of poverty	*Of course, I emphasise abstinence … we have to emphasise it, because when we do this, I’m sure we will have, eh, a better living population, hmm? … There should be an end of suffering… The vicious cycle of poverty should stop… If they manage to go through the academic ladders, of course, they will be financially stable. (Steven, age 29)* [22]
		A dilemma of teaching contraception to students because of the teacher's role and value as a parent that wants to promote abstinence	*It is very difficult for me. As a parent I need to promote abstinence, but as a teacher this curriculum wants me to talk about the importance of using condoms. One topic for example requires us to describe the steps that one has to follow when using a male or female condom. Now, how do I demonstrate such steps to learners who are almost the same age as my child? No, that’s like teaching children to be ‘sex experts’ (IDI, Teacher 7)* [30]
		The female teacher provided sexuality education based on her experience, because she wanted to teach appropriate relationships to her students	*I think I made a mistake, I shouldn’t have got... gotten into a relationship at the university. [...] Perhaps if I had waited... I would not have married the person I married [...]. Because I acted out of... emotions, not out of... reasoning, [...], it was too early for me to do that. Hmm... I believe [...] if I had got the information, I should have acted differently. So, in most of the times my... my information I give to the students is out of my own experience to stop them from... going through with my experience, so even making worse mistakes. Yeah... Because me, I was lucky, [...] I believe... he married me because I got pregnant. [...] That means I married for wrong reasons. So, I believe I talk out of my own experience to the children so that they don’t make the same mistake. (Vivienne, age 43)* [28]
Theme 2: Non-integration of traditional sex education into comprehensive sexuality education	ST 2-1: Challenges of implementation in school the traditional sex education	The rigid structure of the curriculum and supplemental nature of sex education did not encourage teachers to deliver sex education optimally	*The non-flexible nature of the timetable, as well as the non-compulsory nature of sex education activities does not compel teachers to engage to the fullest extent and potential of sex-related discourse and activities. (Head teacher, School Two)* [21]
		Some teachers felt difficulty integrating existing subjects into sex education	*The problem we have is that most teachers do not integrate or infuse sex education into the subjects they teach…I think some of them find it difficult to do it. (School-based Coordinator, School Three)* [21]
		The gap between old and young generations when it comes to conversations about sex	*These days, kids tend … to know about sex … culture has created a gap between the … the young and the old. Simply because the old fear to talk about sex. They always want to keep it as a secret, yet it can never be a secret in today’s society. (Samuel, age 26)* [22]
		Some parents complained to the school principal because a teacher provided a sexual topic (CSE curriculum) to students at school	*Having seen the assignment, which I gave to the learners, parents came in numbers to the school in the morning and demanded to see the headmaster. I was called to attend the meeting. The parents then complained to the headmaster that the initiation ceremony is something special which should not be handled at the school (IDI, Teacher 6)* [30]
	ST 2-2: Consideration for the minorities	Intellectually disabilities learners have some challenges to learn sexuality	*Teaching sexuality in any form is challenging to us educators who teach intellectually challenged learners. I teach the foundation phase learners, and their ages are 6 to 7 years old. They function on a level of 2 to 3 years. Their level of IQ does not allow them to understand fully the context around sexuality, HIVand AIDS (grade 2 teacher, coloured female, 10 to 20 years teaching experience)* [25]
		Children had aware of sexual behavior depending on the level of intellectual disability	*I found that when you start talking about sexuality that some of our children (depending of the level of intellectual disability) become more aware on a negative way, as if the sexual behaviour becomes more upfront (grade 7 teacher, white female, 30 to 40 years teaching experience)* [25]
		For HIV/AIDS education, there is difficult to talk with the HIV positive adolescents	*The other challenge is that we are having difficulty in talking to the HIV positive adolescents. We do not have any approach or skills on how to take them aside and talking to them is difficult. As a result, some of them leave school and get married to healthy partners further spreading the scourge. (Kate 28)* [23]
		Homosexuality and gender diversity has not been recognized by the government in the whole country	*Homosexuality is…, since the whole country is against that, I believe that, when somebody tempers, you know, to encourage homosexuality, particularly in school, which is actually at a mission, which is under church mission, definitely, it may end your job (laughing). Yes, so we’re not actually talking and eh telling them that it is good practice to have homo sexuality. (Alex, boys’ boarding school)* [27]
Theme 3: Fostering effective facilitation of CSE among teachers	ST 3-1: Foster effective facilitation of the CSE in the school setting	The problem of ‘old learning aids’ and ‘the need to introduce new one’ for effective facilitation	*…The old nature of teaching and learning aids and the need to introduce new ones will kindle student interest in sex education and foster effective facilitation of sex education. (School-based Coordinator, School Two)* [21]
		The PIASCY (Presidential Initiative on AIDS Strategy to Youth) was well implemented and not occurred the stigmatization	*Even the children who are HIV positive they are also gaining; the stigmatisation is not there anymore and they stay with others freely because of the PIASCY (Presidential Initiative on AIDS Strategy to Youth) that we have. (Kate 28)* [23]
		It's time to introduce sex education by the Ministry of Education and should allow providing education on contraceptive methods generally	*I think it is high time that the ministry of education introduced sex education, so that our young girls and boys can understand what happens. Teaching on sexual education, and even on the use of condoms, should be allowed. (MT7)* [26]
		The comprehensive content such as communication, assertiveness, and decision-making skills that are good for learners are taught repeatedly by the teacher	*I skipped the whole topic on pregnancy prevention. Instead of teaching about condom use, I moved to another topic. I repeated sessions which I thought were good for learners such as communication, assertiveness and decision-making skills (IDI, Teacher 9)* [30]
	ST 3-2: Contents of the traditional sex education should be specified to consist of the CSE	Students from both sexes should be taught about physical changes during puberty to prevent unwanted pregnancy	*In my opinion, girls and boys should be taught on the changes their bodies are undergoing, because some of them are unaware. So, they go out there and they don’t know what happens, and then they end up having unwanted pregnancies. (FT6)* [26]
		Abstinence is seen as the only method to avoid teen pregnancies and STIs	*We have a song about the importance of abstinence, we sing it before we start each session on CSE. I also tell them repeatedly that abstinence is the only method that can help them avoid teen pregnancies and STIs including HIV (IDI, Teacher 14)* [30]
Theme 4: Determining the appropriate age to start sex education	ST 4-1: Recommendation to provide sex education at the beginning of the early adolescent	The teacher concerned about the ways of delivering sex education to children of different age	*Now there are those who are still very young and very innocent… So, for example, if there is a child of 13 years old … and there is another one of 16 in S1 … are [we] going to give this child the same session with the one who is 13? … this child is going to ask you more challenging questions … that a child of 13 might not be comfortable with, might not even understand. (Salimah, age 39)* [22]
		Sexuality education should start at the age, where students start to have their first sexual encounters	*(...) At a later age, from 10 to 14, they change dramatically, they start having their first sexual encounters and I think that is when you should start talking to them about sexuality. (E14)* [29]
	ST 4-2: Lack of educational material based on the development period among students	Need to update the current guidebook because of easy to access sexual information by students’ selves	*So, those who have written the book concerning the subject they should update their books because nowadays children have easy access to much more information when compared to what the books have. The books we are using are now very old. (Lily 48)* [23]
		Lack of guidance on how to provide sexual education within other subjects	*So when I am teaching home economics or religious education, when and how do I introduce sexuality issues in these subjects? This framework does not provide guidance on such issues. This makes teaching very difficult (IDI, Teacher 5)* [30]
		The teacher expressed confusion about ways to integrate learning about relationship in science teaching	*When I am teaching science, how do I bring in issues relating to differences between love, affection and infatuation? It does not work for me (IDI, Teacher 10)* [30]
Theme 5: Roles of stakeholders outside the school	ST 5-1: Lack of collaboration with parents and health workers	Involving parents in counseling for students to enhance behaviors	*My role basically is to form them. Hm? To help them change, behaviour change. That is my role. I do it in many ways. I talk to them. I’ve told you when we needed a reprimand, we do that… when it need counselling, we do that, we involve parents… […] Because… you know, when you are dealing with discipline, changing somebody’s behaviour is not simple. (Male, age 37)* [24]
		Health personnel people should be involved because they know how to tell the children the detailed things	*I think besides teachers the health personnel people should be involved because for them they know how to tell the children the things in details; with us, we simply read from the books. (Kate 28)* [23]
		Some topics could be taught not only by teachers but also by health workers from outside	*Some topics can be taught by teachers and other topics can be taught by people outside the school such as health workers or community health workers (IDI, Teacher 7)* [30]
		The teacher expresses the lack of training in CSE to teach it effectively to students	*The headmasters attended a 2 days training in CSE, and then they briefed teachers in schools on CSE for only one to 2 h. So how do you expect us to effectively teach? (IDI, Teacher 2)* [30]
	ST 5-2: Unclear roles of teachers in CSE implementation	As community members, teachers have roles to support and protect students	*As the member of the community also, teachers play role in making people aware on what is good and what is wrong. And again if there are problems with students, we support them as much as we can, for example, like early marriage, abduction, *etc*., by communicating with police on conditions that students reported to us. (D20, school2, teacher3, male)* [20]
		The teacher hoped to complete academic achievement without unwanted pregnancy	*Because to a young girl, when you get involved into sex, you’re still school-going, you need to fulfil your goal, you need to complete your education, and now you are pregnant, there is a way you become stuck and your goals are not meant to be achieved as expected. (Beatrice, age 35)* [22]
	ST 5-3: Positive impact of the in-service training	Teacher training had a positive impact to change the behavior and confidence of teacher self	*First of all, the training changed my behaviour, before that I have no confidence to discuss about this issue, because of the culture I came through. But now I discuss freely with my children and I teach my neighbours about discussing freely with young children about sexuality issues, and I see the change. (D19, school2, teacher2, female)* [20]
		Integrated in-service training for teachers of adolescent reproductive health was provided	*We have what we call enhanced SHEP (school health education program) and under this, we train all teachers in the school on ARH (adolescent reproductive health) through workshops and seminars at the district assembly and sometimes in schools, and by doing this we equip them with the knowledge and skills to impart them in the classrooms…Because we are doing integration and infusion we train all teachers to infuse sex education in the classrooms and also train School Health Education coordinators to handle clubs in the schools. (Municipal School Health Education Coordinator)* [21]
		The training program by UNICEF was targeted at teachers, headteachers, and students in all the school community	*UNICEF gives training to all the school community in three sessions for teachers, headteachers, and students. So, no teacher can have any excuse when asked to support the school based coordinator for help on sex education issues such as the School Health Club. (School-based Coordinator, School One)* [21]
	ST 5-4: School-based CSE program	School Health Education Program (SHEP) included the content of sexuality education which focused on all teachers, headteachers, and students at the district level	*We (teachers) were trained at workshops on the SHEP program and sex education was part of the training’ (School-based Coordinator, School Two) “…teachers are very supportive and make work less difficult…I think this is because all teachers, headteachers, and students partake in training activities at the district level. (School-based Coordinator, School Three)* [21]
		The manuals and materials by UNICEF are used currently, but it's necessary to bring a new one to enhance teaching and learning under e-SHEP	*The manuals and materials given to us during the initial training by UNICEF are what we are still using (elaborating by showing the researcher an old manual and a leaflet on e-SHEP). I think new ones should be brought in to enhance teaching and learning under e-SHEP. (School-based Coordinator, School Three)* [21]
		The PIASCY (Presidential Initiative on AIDS Strategy to Youth) of the school club had a positive impact on students	*In our school, PIASCY is still going on well and the children like to participate in it a lot. There is a club, a PIASCY club for the children and they are the ones who mobilise their friends when it is time for them, but we the teachers just give them instructions that the theme for this week is like this and they formulate the play or role play or recite a poem. So, it’s healthy and sometimes the peers teach one another. (Kate 28)* [23]

#### Theme 1: Hesitancy in talking about sex education among teachers due to the cultural and religious context

Some of the teachers were hesitant to talk to students at school about sexual and reproductive health (SRH). This is because SRH is still a sensitive topic, and it might be necessary to show what the right way is as an option for students. It seems difficult to facilitate interest in CSE among teachers [20, 21]. The teacher is afraid that sex education will promote ‘sexual awakening’ among children and may encourage student sexual behavior. Moreover, some of the parents have not yet acknowledged talking about sexuality at school, because they felt that sex education fosters ‘sexual awakening’ among children [25]. The teacher also expressed concerns on losing their moral authority as a professional educator in front of students, which results in a loss of control over students. Furthermore, the relationship between students and educators is not easy to develop since teachers are perceived as disciplinarians [24, 29].

Many teachers mentioned that providing information on contraceptive methods depends on teachers’ perceptions and experiences. Teaching sex education and demonstrating contraceptive methods in school remain taboo due to being culturally inappropriate [22, 30]. Sexuality education is still inhibited due to both cultural and religious backgrounds, and these multiple pressures might impact the educator [22]. The main message of sex education is abstinence, and the teacher hopes to share the belief of the benefits of abstinence education with students [22, 28, 30]. Conflict occurs when teachers have to teach contraceptive methods to students as a teacher at school, while they must teach abstinence to children as a parent at home [28].

#### Theme 2: Non-integration of traditional sex education into comprehensive sexuality education

The challenge of implementation in school is that majority of the official subjects on traditional sex education have not been integrated into the official curriculum [21]. In addition, some teachers are afraid that students tended to easily learn about sex recently; thus, there is a gap between teachers and students [22]. Furthermore, the parents complained to the school principal, because a teacher provided a sexual topic (as a CSE curriculum) to students at school [30]. Regarding consideration of minorities, three main points were identified: intellectually disabled learners; HIV-positive adolescents; and homosexuals [23, 25, 27].

#### Theme 3: Fostering effective facilitation of CSE among teachers

Teachers reported positive motivation toward fostering the effective facilitation of CSE in the school setting. One of the teachers taught comprehensive content, such as communication, assertiveness, and decision-making skills. Therefore, both old and new types of integrated sex education could enhance the effective facilitation of sex education in schools [21, 23, 26, 30]. In addition, the contents of traditional sex education should be included in CSE. Abstinence, which is discussed in traditional sex education, can help avoid teen pregnancies and STIs, including HIV and should, therefore, be included in CSE [26, 30].

#### Theme 4: Determining the appropriate age to start sex education

Some teachers recommended providing sex education at the beginning of early adolescence***,*** since almost all adolescents start to talk about sexuality during this period [22, 29]. Moreover, there is a challenge to the lack of educational material aimed at this developmental period. Teaching CSE to this cohort is challenging, since the current framework has not yet identified its moderation throughout lectures on home economics or religious education. In addition, students can easily access information on sexuality; thus, the need to update the current guidebook was also identified [23, 30].

#### Theme 5: Roles of stakeholders outside the school

Parties outside the school were involved in the implementation of the CSE. Teachers recommended involving parents in counseling for students [24]. Through this involvement, parents will start talking about sex education with their children and enhance behaviors for promoting CSE [24]. The teacher’s role is to involve parents to form students’ behavior and have a role to support students as community members. The teachers hoped for students to complete their school education without unwanted pregnancy [22]. It is also reported that the health sector is responsible for some of the health education on CSE [23, 30]. It is common for health center personnel to directly conduct health education on certain health issues in schools on behalf of teachers.

In-service training by UN agencies positively impacted teachers and also influenced teachers to change their own behavior [20, 21]. The training program by the United Nations International Children’s Emergency Fund (UNICEF) was targeted at teachers, head-teachers, and students in the school community and was enhanced [21]. It was indicated that school-based CSE programs are essential to strengthen CSE. This is because the Presidential Initiative on AIDS Strategy to Youth (PIASCY) school club in Uganda had a positive impact on students [23].

## Discussion

This qualitative systematic review aimed to describe the conflicts experienced by teachers in the implementation of CSE in schools. Furthermore, the study aimed to identify the causes of conflict among teachers in the implementation of CSE.

This study identified the following findings regarding the implementation of CSE in the school setting. First, it was indicated that a transition from traditional sex education to comprehensive sexuality education (CSE) is needed. Second, it should be clear that an appropriate stage and consideration of sexual minorities for implementing CSE. Third, the teacher’s role in implementing sex education has not been identified; thus, the roles of teachers and other institutions should be clarified. Five themes on the causes of the conflict emerged from the thematic meta-synthesis: 1) Hesitancy in talking about sex education among teachers due to the cultural and religious context; 2) Non-integration of traditional sex education into comprehensive sexuality education; 3) Fostering effective facilitation of CSE among teachers; 4) Determining the appropriate age to start sex education; and 5) Roles of stakeholders outside the school.

As shown in Table 6, the five themes described the characteristics of conflicts based on the synthesized studies’ findings. The first theme, “Prohibitions on sexuality education”, enumerated the reasons why sexuality education is still prohibited. Notably, sub-theme 1–5 showed the association between sexuality education and religious backgrounds. The theme “Commonality of the conflicts” indicated common challenges identified by most of the included studies. The third theme, “Diversity of the conflicts”, showed the diversity of the conflicts throughout this systematic review and qualitative meta-synthesis. Finally, the “Suggestions for future promotion of school-based sexuality education”, describes recommendations which should be overcome to promote school-based sexuality education.

**Table 6:** Overview of the analysis of conflicts

Characteristics of the conflicts	Analytical theme	Sub-theme (ST)
Prohibitions on sexuality education	Theme 1: Hesitancy in talking about sex education among teachers due to the cultural and religious context	ST 1-1: The topic of Sexual and Reproductive Health (SRH) is a still taboo
		ST 1-2: Concerns that sexuality education might encourage unhealthy curiosity and experimenting sexual behaviors
		ST 1-3: Concerns/Fear that teacher would lose moral authority and control over the students
		ST 1-4: Providing information on contraceptive methods is depending teachers’ perceptions and experiences
		ST 1-5: Religious and cultural norms as inhibitor in implementing sex education
		ST 1-6: Abstinence education as inhibitor of CSE implementation
Commonality of the conflicts	Theme 2: Non-integration of traditional sex education into comprehensive sexuality education	ST 2-1: Challenges of implementation in school the traditional sex education
		ST 2-2: Consideration for the minorities
Diversity of the conflicts	Theme 3: Fostering effective facilitation of CSE among teachers	ST 3-1: Foster effective facilitation of the CSE in the school setting
		ST 3-2: Contents of the traditional sex education should be specified to consist of the CSE
Suggestions for future promotion of school-based sexuality education	Theme 4: Determining the appropriate age to start sex education	ST 4-1: Recommendation to provide sex education at the beginning of the early adolescent
		ST 4-2: Lack of educational material based on the development period among students
	Theme 5: Roles of stakeholders outside the school	ST 5-1: Lack of collaboration with parents and health workers
		ST 5-2: Unclear roles of teachers in CSE implementation
		ST 5-3: Positive impact of the in-service training
		ST 5-4: School-based CSE program

The transition from traditional sex education to CSE is crucial to foster effective CSE at school. The integration of traditional sex education and CSE can influence effective facilitation among teachers [21, 23, 26, 30]. This is because CSE implementation still depends on teachers’ experience and perception due to the lack of an official curriculum or guideline. Policies related to the abstinence approach have still prohibited the demonstration of contraceptive methods in sex education [10]. It was reported that the provision of information about contraception influenced immoral and health-compromising sexual behaviors among students due to an abstinence message [31]. Thus, as an influence of the abstinence approach, there is a need to emphasize the provision of not only the physical aspect of sex education but also the promotion of moral education and appropriate relationships among students. It was reported that if teachers understand only the biological or physical components of sex education, such as the differences between male and female bodies, the mechanism of pregnancy, and the prevention of STDs, teachers might be afraid that CSE would have a negative effect of encouraging students to engage in promiscuous sexual behavior. Conversely, if teachers understand the children’s human rights component of comprehensive sexuality education, they will be able to understand that it promotes independent understanding of sexuality among children, rather than the perceived negative effects [32, 33].

In terms of the dissemination of CSE, this study reveals the need to consider sexual minorities among adolescents in society, as well as the appropriate age to start CSE, because the appropriate age has not been specified and some of the teachers felt conflicted about the period of starting sex education still being delayed. The relevant literature includes substantial evidence to support that sex education is most effective when it begins early, before young people start engaging in sexual activity [32]. On the other hand, it was reported that LGBTQ students still faced a hostile environment in school, because they felt that they routinely heard anti-LGBTQ language and experienced victimization and discrimination [9, 34]. Hence, to solve these challenges, CSE should be provided at an appropriate stage, with consideration of sexual diversity and sexual minorities in schools.

Although multiple subjects should be covered by educators, one study that was selected in this review noted that the teacher’s role in implementing CSE has not been officially identified [21]. Since underlying factors included the incompatibility of CSE with the traditional culture and religious norms in each country, teachers were regarded as having a parental role at schools, with an obligation to teach students to recognize responsibility or morals [30]. A study conducted in six Southern African Countries showed that CSE was commonly provided by life orientation teachers or class teachers in schools [35]. Moreover, this study reported that learners (students) preferred to be taught CSE by family members, such as parents or grandparents, and it indicated the importance of the family role in the implementation of CSE. Especially, in low- and middle-income countries, the provision of health services is mostly covered by the private sector and there are international non-government organizations that specialize in sexual and reproductive health (SRH) policy [36]. Therefore, CSE should not only be taught by teachers in schools, but should also involve parents, the private sector, and health workers in the community.

Complaints by parents to school principals due to the provision of sexual topics to students in schools was reported in rural Zambia [30]. Considering this case, enhancing the leadership and connections of the school principals with parents in the community should also be explored. According to a previous study, it was considered that support by school principals set a foundation for cultural change and working for a robust school environment, and training for principals might foster leadership and family engagement in the community [37]. Moreover, the strengthening of partnership-building as one of the core educational provisions between parents and educators would support students in accessing necessary information [38]. Roberts revealed that the role of principals as a spokesperson, negotiator, and coordinator within the school environment is crucial in implementing comprehensive school health [39]. Therefore, school principal leadership might be recognized as a crucial role in linkage with parents in the community, which is needed to change the school culture and perceptions toward CSE.

The results of the thematic meta-analysis also emphasized the need for further research in other regions, since the findings were synthesized based on findings from Africa and Spain. The CSE status report in 2021 recommended three points for CSE implementation in countries worldwide: *1) Clear mandates and budgets to ensure implementation of policies and programs that support the availability of good quality comprehensive sexuality education for all learners*; *2) Invest in quality curriculum reform and teacher training*; and *3) Strengthen monitoring of the implementation of CSE* [6]. These recommendations address the causes of the conflict among teachers which were identified in the thematic meta-synthesis. Furthermore, the findings of the meta-synthesis indicated a necessity for further research to explore the implementation of school-based sexuality education.

The studies included in the meta-synthesis mostly reflected the experience of African countries and thus may have missed the context in other regions. For example, the Asia–Pacific region, which is home to more than half of the 1.8 billion young people of 10–24 years of age [40], still has diverse socio-economic and cultural factors that can negatively influence access to sexual and reproductive health and rights services and the provision of CSE [41].

The findings of this thematic meta-synthesis also strongly reflected the Christian context. Religions have different norms and beliefs; thus, there is a need to explore the experience in countries with other religions, including Islam. A systematic review that focused on Muslim women in 2020 indicated that Islamic norms still tended to prohibit sexual and reproductive health education and access to relevant information [42]. Alomair et al. recommended that an intervention for Muslim people may contribute to overcoming barriers to reproductive health education and services to improve knowledge [42]. Hence, further research on implementing CSE in Asian regions and other religious contexts could be enhanced.

The present study was associated with two limitations. The first is that it only included English-language papers. Since the search focused on keywords and databases that reflect peer-reviewed sex education and sexual health literature, some of the research published outside of this literature may not have been fully represented. Therefore, the search strategy limited the ability to evaluate all of the existing literature. The second limitation is that the systematic approach was not exhaustive and may not have yielded the full range of evidence and findings, and it was assumed that some relevant papers were not included due to the search strategy or selection database. Thus, the approach should be enhanced by searching a broad selection of databases and screening reference lists of all included papers to conduct a gray literature review, or by conducting a hand search.

## Conclusion

This qualitative systematic review and thematic meta-synthesis highlighted several conflicts among teachers in the implementation of CSE. Despite the teachers providing sex education, the education they provide has not yet transformed from traditional sex education to CSE. The study findings also emphasize the need to identify the role of teachers in CSE implementation as family members at schools. Furthermore, it has indicated the need to clarify how collaboration with parents, the private sector, and health workers in the community can be done. This thematic meta-synthesis also strongly reflected the context of Christianity in Europe and Africa; thus, further research on the religious context in other regions is needed.

## Data Availability

Not applicable.

## Abbreviations

CSE: Comprehensive sexuality education
ENTREQ: Enhancing Transparency in Reporting the Synthesis of Qualitative Research
PRISMA: Preferred Reporting Items for Systematic Reviews and Meta-Analyses
SRH: Sexual and reproductive health
UNESCO: United Nations Education, Scientific and Cultural Organization
WHO: World Health Organization

## References

[1] World Health Organization. The physical school environment: an essential component of a health-promoting school. Geneva: World Health Organization; 2004. https://apps.who.int/iris/handle/10665/42683.

[2] Xu T, Tomokawa S, Gregorio ER, Mannava P, Nagai M, Sobel H (2020). School-based interventions to promote adolescent health: a systematic review in low and middle-income countries of WHO Western Pacific Region. PLoS ONE.

[3] World Health Organization (2017). Global accelerated action for the health of adolescents (AA-HA!): Guidance to support country implementation.

[4] United Nations Education, Scientific and Cultural Organization UNESCO (2018). International technical guidance on sexuality education: an evidence-informed approach, revised edition.

[5] United Nations Education, Scientific and Cultural Organization (UNESCO) (2015). Emerging evidence, lessons and practice in comprehensive sexuality education: a global review.

[6] United Nations Education Scientific and Cultural Organization (UNESCO) (2021). The journey towards comprehensive sexuality education: global status report.

[7] Ngabaza S, Shefer T, Macleod CI (2016). “Girls need to behave like girls you know”: the complexities of applying a gender justice goal within sexuality education in South African schools. Reprod Health Matters.

[8] Vanwesenbeeck I, Westeneng J, de Boer T, Reinders J, van Zorge R (2016). Lessons learned from a decade implementing comprehensive sexuality education in resource poor settings: the World Starts With Me. Sex Educ.

[9] Goldfarb ES, Lieberman LD (2021). Three decades of research: the case for comprehensive sex education. J Adolesc Health.

[10] Leung H, Shek DTL, Leung E, Shek EYW (2019). Development of contextually-relevant sexuality education: lessons from a comprehensive review of adolescent sexuality education across cultures. Int J Environ Res Public Health.

[11] Chavula MP, Zulu JM, Hurtig AK (2022). Factors influencing the integration of comprehensive sexuality education into educational systems in low- and middle-income countries: a systematic review. Reprod Health.

[12] Elgoibar P, Euwema M, Munduate L (2017). Conflict management. Oxford Res Encycl Psychol.

[13] Lewin K (1935). A dynamic theory of personality.

[14] Noyes J, Booth A, Cargo M, Flemming K, Harden A, Harris J, et al. Chapter 21: Qualitative evidence. In: Higgins JPT, Thomas J, Chandler J, Cumpston M, Li T, Page MJ, et al. (editors). Cochrane Handbook for Systematic Reviews of Interventions version 6.3 (updated February 2022). Cochrane, 2022. https://training.cochrane.org/handbook.

[15] Tong A, Flemming K, McInnes E, Oliver S, Craig J (2012). Enhancing transparency in reporting the synthesis of qualitative research: ENTREQ. BMC Med Res Methodol.

[16] Butler A, Hall H, Copnell B (2016). A guide to writing a qualitative systematic review protocol to enhance evidence-based practice in nursing and health care. Worldviews Evid Based Nurs.

[17] Page MJ, McKenzie JE, Bossuyt PM, Boutron I, Hoffmann TC, Mulrow CD (2021). Preferred reporting items for systematic reviews and meta-analyses: the PRISMA Statement. BMJ.

[18] The Joanna Briggs Institute (2014). Joanna Briggs Institute Reviewer’s Manual: 2014 Edition.

[19] Thomas J, Harden A (2008). Methods for the thematic synthesis of qualitative research in systematic reviews. BMC Med Res Methodol.

[20] Le Mat MLJ, Miedema EAJ, Amentie SA, Kosar-Altinyellken H (2021). Moulding the teacher: factors shaping teacher enactment of comprehensive sexuality education policy in Ethiopia. Compare.

[21] Ocran BE (2021). Teacher approaches, attitudes, and challenges to sexuality education: a case study of three junior high schools in Ghana. Afr J Reprod Health.

[22] de Haas B, Hutter I (2019). Teachers’ conflicting cultural schemas of teaching comprehensive school-based sexuality education in Kampala, Uganda. Cult Health Sex.

[23] Achora S, Thupayagale-Tshweneagae G, Akpor OA, Mashalla YJS (2018). Perceptions of adolescents and teachers on school-based sexuality education in rural primary schools in Uganda. Sex Reprod Healthc.

[24] de Haas B, Hutter I (2020). Teachers’ professional identities in the context of school-based sexuality education in Uganda—a qualitative study. Health Educ Res.

[25] Louw JS (2017). A qualitative exploration of teacher and school staff experiences when teaching sexuality education programmes at special needs schools in South Africa. Sex Res Soc Policy.

[26] Håkansson M, Super S, Oguttu M, Makenzius M (2020). Social judgments on abortion and contraceptive use: a mixed methods study among secondary school teachers and student peer-counsellors in western Kenya. BMC Public Health.

[27] Rijsdijk LE, Bos AE, Lie R, Leerlooijer JN, Eiling E, Atema V (2014). Implementation of The World Starts With Me, a comprehensive rights-based sex education programme in Uganda. Health Educ Res.

[28] de Hass B, Hutter I (2022). Teachers’ personal experiences of sexual initiation motivating their sexuality education messages in secondary schools in Kampala, Uganda. Sex Educ.

[29] Plaza-Del-Pino FJ, Soliani I, Fernández-Sola C, Molina-García JJ, Ventura-Miranda MI, Pomares-Callejón MÁ (2021). Primary school teachers’ perspective of sexual education in Spain. A qualitative study. Healthcare (Basel).

[30] Zulu JM, Blystad A, Haaland MES, Michelo C, Haukanes H, Moland KM (2019). Why teach sexuality education in school? Teacher discretion in implementing comprehensive sexuality education in rural Zambia. Int J Equity Health.

[31] Kirby, D. Emerging Answers 2007: Research Findings on Programs to Reduce Teen Pregnancy and Sexually Transmitted Diseases. Washington, DC,: National Campaign to Prevent Teen and Unplanned Pregnancy; 2007. https://powertodecide.org/sites/default/files/resources/primary-download/emerging-answers.pdf. Accessed 12 October 2022.

[32] Thammaraksa P, Powwattana A, Lagampan S, Thaingtham W (2014). Helping teachers conduct sex education in secondary schools in Thailand: overcoming culturally sensitive barriers to sex education. Asian Nurs Res.

[33] Ramírez-Villalobos D, Monterubio-Flores EA, Gonzalez-Vazquez TT, Molina-Rodríguez JF, Ruelas-González MG, Alcalde-Rabanal JE (2021). Delaying sexual onset: outcome of a comprehensive sexuality education initiative for adolescents in public schools. BMC Public Health.

[34] Kosciw JG, Greytak EA, Zongrone AD, Clark CM, Truong NL (2018). The 2017 National School Climate Survey: the experiences of lesbian, gay, bisexual, transgender, and queer youth in our nation’s schools.

[35] Chawhanda C, Ogunlela T, Mapuroma R, Ofifinni O, Bwambale MF, Levin J (2021). Comprehensive sexuality education in six Southern African countries: perspectives from learners and teachers. Afr J Reprod Health.

[36] Peters DH, Mirchandani GG, Hansen PM (2004). Strategies for engaging the private sector in sexual and reproductive health: how effective are they?. Health Policy Plan.

[37] Smith TE, Reinke WM, Herman KC, Sebastian J (2021). Exploring the link between principal leadership and family engagement across elementary and middle school. J Sch Psychol.

[38] Hands C (2005). It’s who you know “and” what you know: the process of creating partnerships between schools and communities. Sch Community J.

[39] Roberts E, McLeod N, Montemurro G, Veugelers PJ, Gleddie D, Storey KE (2016). Implementing comprehensive school health in Alberta, Canada: the principal’s role. Health Promot Int.

[40] United Nations Population Fund (2020). My body is my body, my life is my life: Sexual and reproductive health and rights of young people in Asia and the Pacific.

[41] UNFPA, UNESCO, IPPF. Learn. Protect. Respect. Empower. The status of comprehensive sexuality education in Asia and the Pacific: A summary review 2020.UNFPA: 2021.

[42] Alomair N, Alageel S, Davies N, Bailey JV (2020). Factors influencing sexual and reproductive health of Muslim women: a systematic review. Reprod Health.

